# Factors predicting a home death among home palliative care recipients

**DOI:** 10.1097/MD.0000000000008210

**Published:** 2017-10-13

**Authors:** Ming-Chung Ko, Sheng-Jean Huang, Chu-Chieh Chen, Yu-Ping Chang, Hsin-Yi Lien, Jia-Yi Lin, Lin-Chung Woung, Shang-Yih Chan

**Affiliations:** a Department of Urology, Taipei City Hospital, Taipei City; b Department of Health Care Management, National Taipei University of Nursing and Health Sciences, Taipei City; c School of Medicine, Fu-Jen Catholic University, New Taipei City; d Superintendent Office, Taipei City Hospital, Taipei City; e Department of Surgery, National Taiwan University, Taipei City; f Center of Quality Management, Taipei City Hospital, Taipei City; g Cross-Strait Medical and Management Communication Center, Taipei City Hospital, Taipei City; h Administrative Center, Ministry of Health and Welfare Taipei Hospital, New Taipei City; i Department of Cardiology, Taipei City Hospital, Taipei City, Taiwan.

**Keywords:** home care, home death, palliative care, place of death

## Abstract

Awareness of factors affecting the place of death could improve communication between healthcare providers and patients and their families regarding patient preferences and the feasibility of dying in the preferred place.

This study aimed to evaluate factors predicting home death among home palliative care recipients.

This is a population-based study using a national representative sample retrieved from the National Health Insurance Research Database. Subjects receiving home palliative care, from 2010 to 2012, were analyzed to evaluate the association between a home death and various characteristics related to illness, individual, and health care utilization. A multiple-logistic regression model was used to assess the independent effect of various characteristics on the likelihood of a home death.

The overall rate of a home death for home palliative care recipients was 43.6%. Age; gender; urbanization of the area where the patients lived; illness; the total number of home visits by all health care professionals; the number of home visits by nurses; utilization of nasogastric tube, endotracheal tube, or indwelling urinary catheter; the number of emergency department visits; and admission to intensive care unit in previous 1 year were not significantly associated with the risk of a home death. Physician home visits increased the likelihood of a home death. Compared with subjects without physician home visits (31.4%) those with 1 physician home visit (53.0%, adjusted odds ratio [AOR]: 3.23, 95% confidence interval [CI]: 1.93–5.42) and those with ≥2 physician home visits (43.9%, AOR: 2.23, 95% CI: 1.06–4.70) had higher likelihood of a home death. Compared with subjects with hospitalization 0 to 6 times in previous 1 year, those with hospitalization ≥7 times in previous 1 year (AOR: 0.57, 95% CI: 0.34–0.95) had lower likelihood of a home death.

Among home palliative care recipients, physician home visits increased the likelihood of a home death. Hospitalizations ≥7 times in previous 1 year decreased the likelihood of a home death.

## 1 Introduction

A home death is gradually considered as an important indicator of quality of end-of-life care^[1]^ because most end-staged ill patients worldwide would like to die at home.^[2–5]^ Studies have demonstrated that the contentment of end-of-life care is improved if patients die in their preferred place.^[6,7]^

According to a notional model proposed by Gomes and Higginson,^[8]^ place of death was decided by multiple factors that can be classified into 3 main categories: illness, individual, and environment. Illness-related factors include type of disease, level of disability, and so on. Individual factors include sociodemographic feature and patients’ preferences regarding place of death.^[8]^ Environmental-related factors include health care supply (home care, hospital bed accessibility, and hospital admissions); social support (network of social support, the preferences of care-providers); and macrosocial factors (historical trends).^[8]^

The demands of end-staged ill patients are distinct and some places of death may be more suitable for certain patients than others.^[9]^ The place of death, and a home death particularly, is sometimes regarded as an important indicator of the quality of end-of-life care,^[10]^ however certain factors of place of death could be more modifiable than others. Comprehension of factors affecting the place of death could not only improve communications between health care providers and patients and their families with regard to patient preferences and the feasibility of dying in the preferred place but also inform policy decisions aimed to improve patients’ likelihood of dying in their preferred place of death.^[7]^

Palliative care was introduced in Taiwan by local religious hospitals in the mid-1980s.^[11]^ Taiwan National Health Insurance (NHI) has provided coverage for home palliative care program since 1996. Taiwan passed the “The Hospice Palliative Medical Act” (Natural Death Act) and Taiwan NHI began to provide coverage for inpatient palliative care program in 2000. Taiwan NHI has expanded the indications of hospice care from patients with advanced cancer or amyotrophic lateral sclerosis to those with other terminal conditions requiring comprehensive care since September 2009.^[12]^

Some previous studies, in Taiwan, reported factors associated with a home death among terminally ill patients however most of them included subjects in general population.^[13,14]^ To the best of our knowledge there are scarce studies on factors predicting a home death among home palliative care recipients in Taiwan. To elucidate factors predicting a home death among home palliative care recipients, this study aimed to evaluate the association between a home death and various characteristics related to illness, individual, and health care utilization using a nationally representative sample retrieved from the National Health Insurance Research Database (NHIRD).

## 2 Methods

### 2.1 Study design and data source

This is a population-based study using data from NHIRD, which is provided by Taiwan's National Health Research Institutes. Taiwan launched a single-payer NHI program on March 1, 1995. As of 2014, 99.9% of Taiwan's people were enrolled.^[15]^ For following up a representative group of the population longitudinally, NHIRD contains “cohort datasets” including claims data randomly sampled in year 2000, 2005, and 2010, from all beneficiaries.^[16]^ In our study, we used Longitudinal Health Insurance Database 2005 (LHID2005). LHID2005 contains all registry and claim data of 1 million subjects randomly sampled in year 2005. The registration data of all people who were beneficiaries of the Taiwan NHI program during the period of January 1, 2005 to December 31, 2005 were drawn for random sampling. New claim data of the cohort would be released every year. According to NHIRD, there was no significant difference in the gender distribution between the patients in the LHID2005 and the original NHIRD.^[16]^

After ethical approval from the institution review board of Taipei City Hospital the annual Ambulatory Care Expenditure by Visits (ACEV) file of year 2009–12 from LHID2005 was analyzed in this study. The ACEV provides information on the dates of the visits, up to 3 diagnoses, encrypted identification numbers of the patients and attending physicians, the sexes and dates of birth of the patients, and the codes of medical facilities.^[17]^ In addition, the ACEV has various codes for home palliative care offered by physician, nurses, or other health care professionals. Codes of physician fees for emergency care can be used to identify emergency department visits in 1 year before initiation of home palliative care. The state-run NHI administration performs expert reviews on a random sample of every 50 to 100 ambulatory and inpatient claims in each hospital and clinic on a quarterly basis to ensure the accuracy of the claims data. False reports of diagnosis receive severe penalty from the state-run NHI administration.^[18]^ Some previous studies reported that the NHIRD was a valid resource for population research. One study by Cheng et al compared discharge diagnoses of acute myocardial infarction listed in the NHIRD with those in the medical records obtained from a medical center in Taiwan. The authors reported the positive predictive value for the diagnosis of acute myocardial infarction was 0.88. The consistency rate for coronary intervention, stenting, and antiplatelet prescription at admission was high, yielding a positive predictive value over 0.90. The consistency rate in comorbidity diagnoses was 95.9% among matched acute myocardial infarction cases.^[19]^ Another study by Cheng et al compared records in the NHIRD with those in 1 medical center to evaluate the validity of the NHIRD for patients with a principal diagnosis of ischemic stroke. Patients hospitalized for ischemic stroke in 1999 were identified from both databases. The authors reported that among the 372 cases identified from the NHIRD, 364 cases (97.85%) were confirmed as ischemic stroke by radiology examination and clinical presentation.^[20]^

Using encrypted individual personal identification number, we were able to interlink all datasets. The information on urbanization of the area where the patients lived was available on Registry of Beneficiaries. The Inpatient Expenditures by Admissions file provides information on destination of patients after being discharged as well as information on previous hospitalizations and admissions to intensive care units in 1 year before initiation of home palliative care. In addition, procedure codes of nasogastric tube insertion, indwelling urinary catheterization, and endotracheal tube insertion could be used to identify their utilization. Information on hospital accreditation levels could be obtained from the Registry for Contracted Medical Facilities.^[17]^

### 2.2 Study participants and outcome measurements

Subjects receiving home palliative care, from 2010 to 2012, were analyzed to evaluate the association between a home death and various characteristics related to illness, individual, and health care utilization. Subjects receiving home palliative care were identified using various codes for home palliative care offered by physicians, nurses, or other health care professionals. In our study we followed the definition by Chiang et al and used the insurance system exit as the proxy for death.^[21]^ Of the 542 subjects receiving home palliative care during study period, 488 encountered a death. For differentiating a hospital death from a home death we used the code for patient destination after being discharged which was available from the Inpatient Expenditures by Admissions file. If a subject had a hospitalization with a destination of death, the patient was categorized as having a hospital death. The remainder of patients was considered as having a home death. The information on illness, including cancer, heart failure, chronic lung disease, chronic liver disease, end-stage renal disease, and neurological disease, was obtained with diagnostic codes. We counted those illnesses only when the subjects had at least 1 hospitalization with the diagnosis or 3 outpatient visits with the diagnosis within 1 year prior to initiation of home palliative care. Of the 542 subjects 523 (96.5%) had a diagnosis of cancer, 25 (4.6%) had a diagnosis of heart failure, 22 (4.1%) had a diagnosis of chronic lung disease, 55 (10.2%) had a diagnosis of chronic liver disease, 0 (0%) had a diagnosis of end-stage renal disease, and 99 (18.3%) had a diagnosis of neurological disease.

### 2.3 Statistical analysis

We calculated rates of a home death according to various characteristics related to illness, individual, and health care utilization. A multiple-logistic regression model was used to assess the independent effect of various characteristics on the risk of a home death. All the statistical analyses were performed using the Statistical Analysis Software (SAS) System, version 9.4 (SAS Institute Inc, Cary, NC). A *P* value <.05 was considered statistically significant.

## 3 Results

The overall rate of a home death for home palliative care recipients was 43.6% (Table 1). Compared with subjects <65 years old (39.2%), those 65 to 74 (41.2%), 75 to 84 (49.4%), and ≥85 years old (43.3%) had higher rates of a home death. Compared with males (40.6%), a higher proportion of females died at home (46.7%). With regard to urbanization of the area where the patients lived, subjects living in rural area had the highest rate of a home death (55.8%), followed by those living in less urbanized area (48.4%), those living in highly urbanized area (47.9%), and those living in moderately urbanized area (37.1%). Regarding illness compared with subjects without cancers (42.9%), those with cancers (43.7%) had a higher rate of a home death. Compared with subjects without neurological diseases (42.6%), those with neurological diseases (49.4%) had a higher rate of a home death. With regard to utilization of home palliative care the rates of a home death for subjects with different total numbers of home visits offered by all health care professionals were similar. Compared with subjects with no physician home visit (31.4%) those with 1 physician home visit (53.0%) or ≥2 physician home visits (43.9%) had higher rates of a home death. The rates of a home death for subjects with 0 to 1, 2, and ≥3 home visits by nurses were 44.6%, 43.7%, and 41.9%, respectively. Compared with subjects with utilization of nasogastric tubes, urinary catheters, or endotracheal tubes (41.5%), those without utilization of these devices had a higher rate of a home death (44.9%). For subjects with emergency department visits 0 times, 1 to 3 times, and ≥4 times in previous 1 year, the rates of a home death were 43.1%, 43.6%, and 44.0%, respectively. Compared with subjects with hospitalization 0 to 6 times (46.4%), those with hospitalization ≥7 times (32.3%) in previous 1 year had a lower rate of a home death. For subjects with and subjects without intensive care unit admission in previous 1 year, the rates of a home death were 48.0%, and 42.6%, respectively. For subjects receiving home palliative care offered by medical centers, regional hospitals and district hospitals, the rates of a home death were 44.4%, 44.4%, and 42.5%, respectively.

**Table 1:**
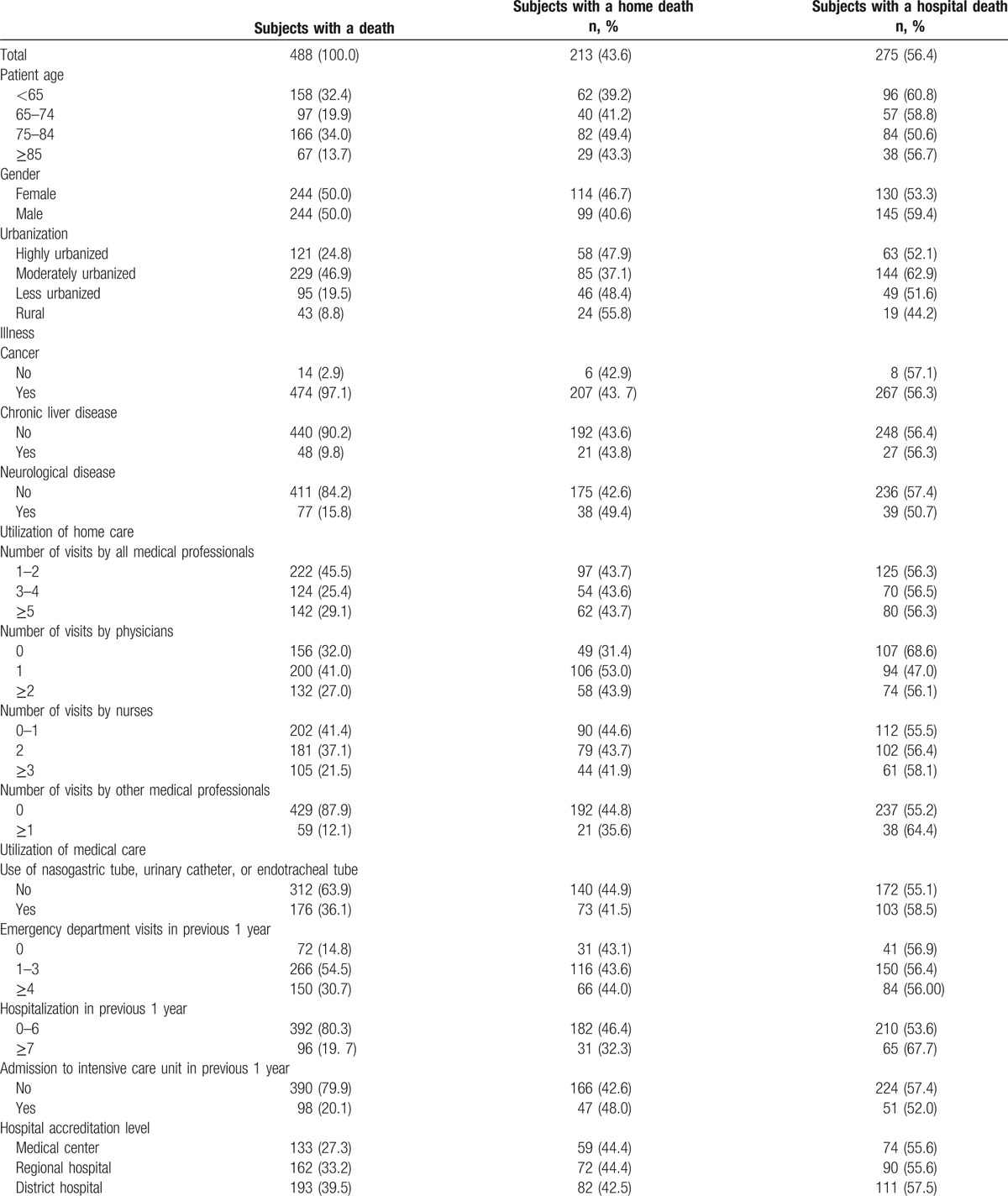
Proportions of a home death according to various characteristics related to illness, individual, and health care utilization.

Table 2 demonstrates the independent effect of various characteristics on the risk of a home death. There was no significant association between patient's age as well as sex and the risk of a home death. There was no significant association between urbanization of the area where the patients lived and the risk of a home death. There was no significant association between patient's illness and the risk of a home death. The total number of home visits offered by all health care professionals and the number of home visits offered by nurses did not have significant effect on the risk of a home death. Home visits offered by physicians increased the likelihood of a home death. Compared with subjects without physician home visits those with 1 physician home visit (adjusted odds ratio [AOR]: 3.23, 95% confidence interval [CI]: 1.93–5.42) and those with ≥2 physician home visits (AOR: 2.23, 95% CI: 1.06–4.70) had higher likelihood of a home death. The utilization of nasogastric tube, urinary catheter, or endotracheal tube and the number of emergency department visits in previous 1 year were not significantly associated with the risk of a home death. Compared with subjects with hospitalization 0 to 6 times in previous 1 year, those with hospitalization ≥7 times in previous 1 year (AOR: 0.57, 95% CI: 0.34–0.95) had lower likelihood of a home death. Admission to intensive care unit in previous 1 year was not significantly associated with the risk of a home death. Hospital accreditation level was not significantly associated with a home death.

**Table 2:**
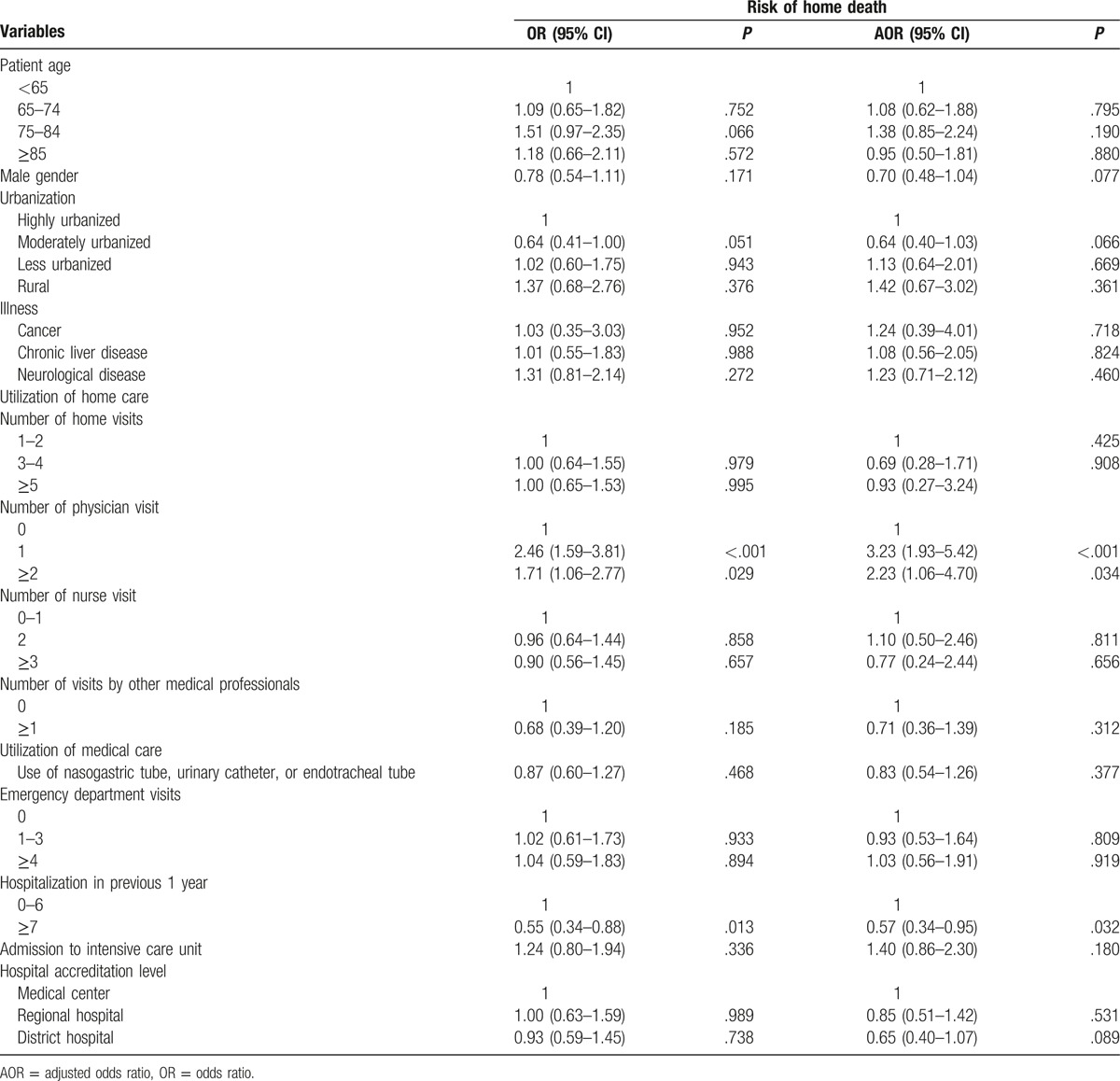
Risk of death at home according to various characteristics related to illness, individual, and health care utilization.

## 4 Discussion

In our study the overall rate of a home death for home palliative care recipients was 43.6%. Home visits by physicians increased the likelihood of a home death. Hospitalization ≥7 times in previous 1 year decreased the likelihood of a home death. Age; sex; level of urbanization where the patients lived; illness; the total number of home visits by all health care professionals; the number of home visits by nurses; utilization of nasogastric tube, urinary catheter, or endotracheal tube; the number of emergency department visits in previous 1 year; and admission to intensive care unit in previous 1 year were not significantly associated with the risk of a home death.

Some previous studies reported different rates of a home death among subjects receiving home palliative care. The study by Brazil et al included participants selected from communities located in south central/western Ontario, Canada, which includes both urban and rural areas. Inclusion criteria for the care recipients included being ≥50 years old, not a resident of a nursing home or assisted living facility, receiving in-home palliative services delivered through a Community Care Access Center. The authors reported that of the 214 home palliative care recipients, the rate of a home death was 56%.^[9]^ Fukui et al had conducted a study to investigate predictors of place of death for Japanese patients with advanced-stage malignant disease in home care settings. Of the 428 home care recipients the rate of a home death was 67%.^[22]^ One retrospective analysis of data on patients referred to the palliative care service located at the National Cancer Centre Singapore reported that of the 842 patients, terminal cancer was the diagnosis for most patients (86%). Two hundred forty-one (29%) died at home.^[23]^ One study in Japan reported that of 4175 subjects receiving home palliative care from nurses, the proportions of causes of death for cancers, cardiovascular diseases, pneumonia, and others were 40%, 12%, 12%, and 36%, respectively. The rate of a home death was 46%.^[24]^ Tang et al used a retrospective cohort from administrative data of 201,201 Taiwanese cancer decedents in the period of 2001–06 and they reported that rates of a home death decreased from 35.7% to 32.4% during study period.^[14]^ Compared with cancer decedents from general population in Taiwan, home palliative care recipients in this study had a higher rate of a home death.

In our study, age was not significantly associated with the risk of a home death among home palliative care recipients. Our result was consistent with some previous studies. The study by Brazil et al reported that, among those receiving in-home palliative services delivered through a Community Care Access Center, age was not associated with a home death.^[9]^ The study by Fukui et al demonstrated that, among home palliative care patients with advanced cancer, there was no significant difference in age between subjects with a home death and those with a hospital death.^[25]^

Regarding the association between urbanization and a home death, the results from previous studies were inconsistent. One retrospective population-based cross-sectional study, involving a total of 697,814 eligible deaths among people ≥65 years old in Taiwan, reported that a home death was associated with lower levels of urbanization.^[13]^ On the contrary, 1 study among terminally ill individuals who received in-home support services in a publicly funded home care system in Canada reported that the level of urbanization was not significantly associated with a home death.^[9]^ The inconsistency in the results may come from the different inclusion criteria of study participants.

In our study, home visits by physicians increased the likelihood of a home death among home palliative recipients. Our results are similar to some previous studies. One cross-sectional nationwide questionnaire survey on home palliative care patients at 1000 randomly selected home care agencies in Japan reported that >3 times of primary nurse consultations with the primary physician during the first week after discharge from hospitals increased the risk of a home death among patients who had cancer with an expected length of survival <6 months.^[25]^ Brazil et al reported that among terminally ill individuals who received in-home support services in a publicly funded home care system, family physician home visits during the care recipients’ last month of life increased the likelihood of a home death.^[8]^ It was also reported that 3 or more GP visits to the patient's home during his or her last 3 months allows more patients to die at home.^[26]^

With regard to the association between home visits by nurses and the risk of a home death, 1 study in Denmark reported that among an unselected population of patients with cancer, home visits by community nurses during the last 3 months before death increased the likelihood of a home death.^[27]^ The study by Neergaard et al reported that, among cancer patients in palliative care at home, involvement of community nurses facilitated a home death.^[26]^ On the contrary, the study by Fukui et al reported that, among home palliative care patients with advanced cancer, number of primary nurse's home visits during first week after discharge (≥3 times) was not associated with a home death.^[25]^ In our study the number of home visits by nurses was not associated with a home death among home palliative care recipients.

In our study increased number of hospitalization decreased the likelihood of a home death. The study by Cardenas-Turanzas et al analyzed 2001 baseline and 2003 follow-up data from the Mexican Health and Aging Study. The participants were adults born before 1951 who completed the baseline interview and died before the follow-up interview and for whom a proxy interview was obtained in 2003. The authors reported that ≥1 hospital admission during the last year of life (vs no admission) decreased the likelihood of a home death.^[28]^ Another study by Fukui et al reported that for Japanese patients with advanced-stage malignant disease in home care settings, the lack of crisis-related rehospitalization during the course of home care was associated with an increased likelihood of a home death.^[22]^ One study with administrative data of 201,201 Taiwanese cancer decedents in the period 2001–06 reported that Taiwanese cancer patients were less likely to die at home if they received care in hospitals.^[14]^ The authors argued that families of the end-staged ill patients may not be capable of taking care of dying patients at home. End-of-life care can be extremely exhausting and expensive for families^[29]^; however, Taiwanese families are not formally reimbursed for the considerable time, effort, and costs they dedicate to caring for terminally ill patients at home.^[14]^ Therefore, families may tend to choose inpatient settings for cancer patients’ end-of-life care.^[5]^

Regarding utilization of medical care, 1 study by Tang et al reported that cancer decedents, from general population, were less likely to die at home if they used life-sustaining treatments (intensive care unit, cardiopulmonary resuscitation, intubation, and mechanical ventilation); however multiple emergency department visits in the last month of life increased the likelihood of a death at home.^[14]^ In our study utilization of nasogastric tube, urinary catheter, or endotracheal tube, the number of emergency department visits in previous 1 year, and admission to intensive care unit in previous 1 year were not significantly associated with the risk of a home death among home palliative care recipients.

Our study has several methodological strengths. First, using claims data of universal national health insurance in clinical research allows easy approach to the longitudinal records of a large sample of geographically disseminated patients and increases the representativeness of the study sample. Second, the NHI dataset provided more accurate and comprehensive information on utilization of medical care among home palliative care recipients which reduced the recall bias. Some limitations in our study should be mentioned. First, using the administrative data we have little information on some important characteristics, including some socioeconomic status, home palliative care recipients’ preference as well as care givers’ preference of place of death and family caregivers’ status and roles, which may confound study results. Second, according to the regulation of Taiwan NHI, only subjects with advanced cancer, amyotrophic lateral sclerosis, or other terminal conditions requiring comprehensive care are eligible for palliative care. However the functional status and the severity of comorbidities of the patient were not available and such information could be essential for characterizing home palliative care recipients.

## 5 Conclusion

Among home palliative care recipients physician home visits increased the likelihood of a home death. Hospitalizations ≥7 times in 1 year before initiation of home palliative care decreased the likelihood of a home death. Awareness of the factors predicting a home death among home palliative care recipients could improve communication between healthcare providers and patients and their families regarding patient preferences and the feasibility of dying in the preferred place.
